# Outcomes and Complications of 2‐Stage Versus 3‐Stage Paramedian Forehead Flaps

**DOI:** 10.1002/ohn.1210

**Published:** 2025-03-10

**Authors:** W. Jack Palmer, Dev Amin, Praneet Kaki, Matt Davis, Eric Fei, Neha Garg, Daniel J. Campbell, Dana Michlin, Khashayar Arianpour, Howard Krein, Ryan Heffelfinger

**Affiliations:** ^1^ Department of Otolaryngology–Head and Neck Surgery Thomas Jefferson University Hospitals Philadelphia Pennsylvania USA; ^2^ Sidney Kimmel Medical College at Thomas Jefferson University Philadelphia Pennsylvania USA

**Keywords:** facial reconstruction, forehead flap, Mohs reconstruction, regional flaps, skin cancer

## Abstract

**Objective:**

This study aims to explore outcomes among 2‐stage paramedian forehead flaps (PFFs), 3‐stage PFFs, and PFFs undergoing accelerated pedicle takedown.

**Study Design:**

A retrospective review.

**Setting:**

A tertiary care institution.

**Methods:**

Patients who underwent PFFs for nasal defects between 2017 and 2022 were identified. Demographic, clinical, and surgical characteristics were compared among groups. Surgical and cosmetic outcomes and revision procedures were evaluated.

**Results:**

Among 52 patients analyzed, 39 underwent 2‐stage PFFs, and 13 underwent 3‐stage PFFs. There were no significant differences in demographics, comorbidities, or surgical risk factors between groups. Three‐stage PFF patients were more likely to have a cartilaginous defect. Postoperative dyspigmentation was seen more frequently in the 3‐stage group; otherwise, there were no significant differences in outcomes. In a subanalysis of 29 patients requiring cartilage grafting, dyspigmentation was again seen more commonly in the 3‐stage group; outcomes otherwise did not favor either group. Within the 2‐stage group, 7 patients underwent accelerated pedicle takedown (≤21 days). No failures were seen with accelerated takedown, including among those who also received cartilage grafting. Overall, accelerated takedown was not associated with poorer surgical or cosmetic outcomes or an increased revision rate compared to standard takedown. Logistic regression did not identify any independent predictors of complication.

**Conclusion:**

Both 2‐ and 3‐stage PFFs are effective tools in midface reconstruction, including when cartilage grafting is required. With 2‐stage PFF, accelerated pedicle takedown is not associated with increased complications in appropriately selected patients.

The paramedian forehead flap (PFF) is an axial, interpolated flap with broad applications in midface reconstruction.^1^ It was initially described as a 2‐stage procedure, including flap elevation, thinning, and inset, followed by pedicle division several weeks later.^2^, ^3^ Advanced techniques in PFF have since emerged. These include the 3‐stage PFF, which involves the transfer of a full‐thickness flap in the first stage, followed by thinning and contouring in an intermediate stage before pedicle division in the final stage.^3^, ^4^ Some favor the 3‐stage method in complex and aesthetically sensitive reconstructions, reporting that the full‐thickness flap maximizes blood flow, can more reliably support cartilage grafts, and can ultimately tolerate more aggressive thinning.^3^, ^4^, ^5^ However, this supposed superiority is not uniformly upheld in the literature, and the longer treatment course has drawbacks.^3^, ^6^ Accelerated pedicle takedown has also emerged as an alternative to the traditional PFF. Proponents assert that dividing the vascular pedicle earlier than the standard 3‐ to 4‐week mark is safe and reduces the time patients must spend in disfiguring intermediate stages.^7^

The present study aims to compare surgical and cosmetic complications among patients undergoing 2‐stage versus 3‐stage PFFs. It also aims to compare these outcomes among patients in the 2‐stage group undergoing accelerated versus standard pedicle takedown. Finally, it aims to identify risk factors associated with complications after PFF.

## Methods

Following Thomas Jefferson University Institutional Review Board approval, a retrospective chart review was conducted for all patients who underwent PFF for facial defects between 2017 and 2022 at a single tertiary care institution. Surgeries were performed by board‐certified facial plastic surgeons. Patients under 18 years, those who received simultaneous free flaps, those with total rhinectomy defects, and those without nasal involvement were excluded. Demographic, clinical, and surgical characteristics were extracted for each patient. Surgical complications, including postoperative hematoma, infection, wound breakdown, necrosis, or fistulization, and cosmetic complications, including the presence of pincushioning, hypertrophic scarring, excess edema, dyspigmentation (including hyperpigmentation, hypopigmentation, and abnormal erythema), trapdoor deformity, or alar notching, as judged by attending facial plastic surgeons at the time of follow‐up, were also recorded. Select characteristics are summarized in the attached tables. “Prior head/neck surgery” and “Prior head/face Mohs” excluded the resection immediately preceding PFF. Defect size is reported as the area of an ellipse with length and width as defined from surgical notes. “Additional reconstruction required” refers to whether an additional technique such as cheek advancement or septal hinge flap was used to address the primary defect.

Patients were grouped by operative technique: 3‐stage PFF (initial surgery, intermediate surgery with pedicle intact, and pedicle takedown), 2‐stage PFF with standard takedown time (initial procedure followed by pedicle takedown >21 days later), and 2‐stage PFF with accelerated takedown (≤21 days later).

Complications noted at any point between the initial surgery and the end of the follow‐up window were included in our analyses. Follow‐up windows are reported as the time between pedicle ligation and the most recent follow‐up. A minority of patients had malignancy recur after pedicle takedown and underwent additional resection; in these cases, the follow‐up window is reported as the time between pedicle division and their re‐resections.

Continuous variables are reported as mean (standard deviation) and categorical variables as frequency (percentage). Chi‐square and Fisher's exact tests were used to compare categorical variables, while Kruskal–Wallis rank‐sum and Wilcoxon rank‐sum tests were used for continuous variables. Separate univariate and multivariable binary logistic regression analyses were performed to identify factors associated with cosmetic complications, surgical complications, or future revision, respectively. The 2‐sided threshold for significance was set at *P* < .05. R Studio Version 2023.03.1 was used for statistical analyses. G*Power Version 3.9.1.6 was used for power analyses.^8^

## Results

Fifty‐two patients met the inclusion criteria. The average age was 70 years. Sixty percent (n = 31) were male and 40% (n = 21) were female. Ninety‐six percent (n = 50) had defects after resection of malignancy, while the remaining 4% (n = 2) had PFFs to reconstruct nasocutaneous fistulae. All patients had defects involving the nose; 23% (n = 12) also had periorbital involvement, and 31% (n = 16) had involvement of other facial subunits. Twenty‐five percent (n = 13) underwent 3‐stage PFF, while 75% (n = 39) underwent 2‐stage PFF. Within the 2‐stage group, 82% (n = 32) were managed with standard‐timeline pedicle division, and 18% (n = 7) were managed with accelerated takedown. Fifty‐six percent of patients (n = 29) received cartilage grafts. All patients with internal nasal defects received vascularized repairs. The mean follow‐up across the cohort was 14 months. There was no significant difference in mean follow‐up between the 3‐stage group (17.6 months), the 2‐stage group with standard pedicle takedown (12.0 months), and the 2‐stage group with accelerated pedicle takedown (16.8 months; *P* = .6).

### Two‐Stage Versus Three‐Stage Groups

Table 1 shows demographic, clinical, and surgical data for the cohort. There were no significant differences in demographics or comorbidities between the 2‐ and 3‐stage groups. Similarly, there were no significant differences in surgical risk factors such as smoking, preoperative or adjuvant radiation, prior head/neck surgery, or regional trauma. Defect size was not significantly different between the 2 groups. Three‐stage PFFs were significantly more likely than 2‐stage PFFs to have a cartilage defect (85%, 44%; *P* = .01).

**Table 1:** Demographics and Comorbidities in 2‐Stage Versus 3‐Stage Groups

Characteristic	Overall (N = 52^a^)	2‐stage (N = 39^a^)	3‐stage (N = 13^a^)	*P*
**Demographics**				
Age	70 (65, 76)	70 (64, 75)	73 (65, 80)	.4
BMI	26.4 (23.7, 29.8)	28.1 (24.8, 30.0)	24.4 (23.0, 28.6)	.2
Gender				.3
Female	21 (40%)	14 (36%)	7 (54%)	
Male	31 (60%)	25 (64%)	6 (46%)	
**Comorbidities**				
Environmental allergies	8 (15%)	4 (10%)	4 (31%)	.10
Chronic rhinosinusitis	2 (3.8%)	0 (0%)	2 (15%)	.06
Obstructive sleep apnea	5 (9.6%)	3 (7.7%)	2 (15%)	.6
Prior cerebrovascular accident	2 (3.8%)	2 (5.1%)	0 (0%)	>.9
Coronary artery disease	11 (21%)	7 (18%)	4 (31%)	.4
Prior myocardial infarction	6 (12%)	4 (10%)	2 (15%)	.6
Congestive heart failure	1 (1.9%)	0 (0%)	1 (7.7%)	.3
Atrial fibrillation	6 (12%)	3 (7.7%)	3 (23%)	.2
Hypertension	34 (65%)	24 (62%)	10 (77%)	.5
Chronic kidney disease	6 (12%)	6 (15%)	0 (0%)	.3
Diabetes	8 (16%)	6 (16%)	2 (15%)	>.9
Prior pulmonary embolism	1 (1.9%)	1 (2.6%)	0 (0%)	>.9
Hepatic dysfunction	4 (7.7%)	2 (5.1%)	2 (15%)	.3
Bleeding/thrombotic disorder	2 (3.8%)	2 (5.1%)	0 (0%)	>.9
Connective tissue disorder	1 (1.9%)	0 (0%)	1 (7.7%)	.3
Anxiety	7 (13%)	5 (13%)	2 (15%)	>.9
Depression	7 (13%)	6 (15%)	1 (7.7%)	.7
Alcohol use	34 (69%)	27 (75%)	7 (54%)	.2
Tobacco use				.10
Current	14 (27%)	12 (31%)	2 (15%)	
Former	26 (50%)	21 (54%)	5 (38%)	
Never	12 (23%)	6 (15%)	6 (46%)	
**Treatments/medications**				
Prior head/neck surgery	12 (23%)	9 (23%)	3 (23%)	>.9
Prior head/face Mohs	11 (21%)	10 (26%)	1 (7.7%)	.3
Prior nasal/facial trauma	4 (8.0%)	3 (8.1%)	1 (7.7%)	>.9
Prior head/neck radiation	8 (15%)	6 (15%)	2 (15%)	>.9
Prior chemotherapy	7 (13%)	4 (10%)	3 (23%)	.3
Adjuvant radiation	13 (25%)	10 (26%)	3 (23%)	>.9
Antiplatelet use	11 (22%)	7 (19%)	4 (31%)	.4
Other anticoagulant use	4 (8.0%)	2 (5.4%)	2 (15%)	.3
Chronic steroid use	1 (1.9%)	1 (2.6%)	0 (0%)	>.9
Immunosuppressant use	3 (5.8%)	2 (5.1%)	1 (7.7%)	>.9
**Defect characteristics**				
Indication				>.9
Defect after malignancy	50 (96%)	37 (95%)	13 (100%)	
Repair facial fistula	2 (3.8%)	2 (5.1%)	0 (0%)	
Defect size, cm^2^	8 (4, 13)	8 (5, 13)	8 (4, 13)	.9
Presence of cartilage defect	28 (54%)	17 (44%)	11 (85%)	**.01**
Presence of internal nasal defect	30 (58%)	20 (51%)	10 (77%)	.11
Additional reconstruction required	25 (48%)	20 (51%)	5 (38%)	.4
Defect involvement				
Nasal	52 (100%)	39 (100%)	13 (100%)	‐
Periorbital	12 (23%)	11 (28%)	1 (7.7%)	.3
Other facial	16 (31%)	11 (28%)	5 (38%)	.5

The bold value indicates the statistical significance of *p* < .05.

^a^ n (%); median (IQR).

Table 2 shows outcomes, including surgical complications, cosmetic complications, and additional/revision procedures for the cohort. There were no total flap failures. Two patients in the 3‐stage group developed partial flap necrosis; one of these patients was ultimately managed in 4 stages due to this complication. No necrosis was encountered in the 2‐stage group; this difference did not meet significance (*P* = .06). There were no other significant differences in surgical complications between groups. Hypertrophic scarring was the most common cosmetic complication, occurring in 18% of patients treated in 2 stages and 15% of 3‐stage patients (*P* > .9). A significant difference was seen in postoperative dyspigmentation, with 4 instances (31%) in the 3‐stage group and 1 (2.6%) in the 2‐stage group (*P* = .01). The remaining cosmetic complications were not significantly more common in either group. Thirty‐seven percent of the total cohort (n = 19) returned for revision in the operating room or office. These revisions included debulking (9 instances), scar revision (8), periorbital reconstruction (including ectropion repair, browlift, and fat transfer) (5), additional local or free flap (4), and dermabrasion (1). These procedures were not distributed asymmetrically between treatment groups. Seventeen percent of the total cohort (n = 9) underwent postoperative steroid injections, and 21% (n = 11) underwent CO_2_ laser resurfacing; these additional procedures were also not significantly associated with either group.

**Table 2:** Outcomes in 2‐Stage Versus 3‐Stage Groups

Outcome	Overall (N = 52^a^)	2‐stage (N = 39^a^)	3‐stage (N = 13^a^)	*P*
**Surgical complications**				
Hematoma	2 (3.8%)	1 (2.6%)	1 (7.7%)	.4
Infection	1 (1.9%)	1 (2.6%)	0 (0%)	>.9
Wound breakdown	3 (5.8%)	3 (7.7%)	0 (0%)	.6
Necrosis	2 (3.8%)	0 (0%)	2 (15%)	.06
Fistula	1 (1.9%)	1 (2.6%)	0 (0%)	>.9
**Cosmetic complications**				
Pincushioning	7 (13%)	3 (7.7%)	4 (31%)	.06
Hypertrophic scarring	9 (17%)	7 (18%)	2 (15%)	>.9
Excess edema	3 (5.8%)	2 (5.1%)	1 (7.7%)	>.9
Dyspigmentation	5 (9.6%)	1 (2.6%)	4 (31%)	**.01**
Trapdoor deformity	1 (1.9%)	1 (2.6%)	0 (0%)	>.9
Alar notching	5 (9.6%)	2 (5.1%)	3 (23%)	.09
**Additional procedures**				
Steroid injection	9 (17%)	6 (15%)	3 (23%)	.7
CO_2_ laser	11 (21%)	10 (26%)	1 (7.7%)	.3
Subsequent revision	19 (37%)	12 (31%)	7 (54%)	.2

The bold value indicates the statistical significance of *p* < .05.

^a^ n (%).

### Cartilage Grafting Subanalysis

Twenty‐nine patients underwent cartilage grafting: 16 in the 2‐stage group and 13 in the 3‐stage group. No significant differences in demographics, comorbidities, surgical risk factors, or defect characteristics were seen between groups in this subpopulation. Table 3 contains an abridged summary of these parameters.

**Table 3:** Demographics and Comorbidities in the 2‐Stage Versus 3‐Stage Cartilage Graft Subgroup

Characteristic	Overall (N = 29^a^)	2‐stage (N = 16^a^)	3‐stage (N = 13^a^)	*P*
**Demographics**				
Age	71 (65, 77)	70 (65, 75)	73 (65, 80)	.6
Gender				.6
Female	14 (48%)	7 (44%)	7 (54%)	
Male	15 (52%)	9 (56%)	6 (46%)	
**Comorbidities**				
Coronary artery disease	7 (24%)	3 (19%)	4 (31%)	.7
Prior myocardial infarction	4 (14%)	2 (12%)	2 (15%)	>.9
Hypertension	20 (69%)	10 (62%)	10 (77%)	.5
Diabetes	6 (21%)	4 (25%)	2 (15%)	.7
Tobacco use				.2
Current	8 (28%)	6 (38%)	2 (15%)	
Former	13 (45%)	8 (50%)	5 (38%)	
Never	8 (28%)	2 (12%)	6 (46%)	
**Treatments/medications**				
Prior head/neck surgery	9 (31%)	4 (25%)	5 (38%)	.7
Prior head/face Mohs	5 (17%)	4 (25%)	1 (7.7%)	.3
Prior nasal/facial trauma	3 (10%)	2 (12%)	1 (7.7%)	>.9
Prior head/neck radiation	3 (10%)	1 (6.2%)	2 (15%)	.6
Adjuvant radiation	8 (28%)	5 (31%)	3 (23%)	.7
**Defect characteristics**				
Defect size, cm^2^	7.9 (3.7, 12.6)	7.7 (4.1, 9.6)	7.9 (3.5, 12.6)	>.9
Presence of internal nasal defect	24 (83%)	14 (88%)	10 (77%)	.6

The bold value indicates the statistical significance of *p* < .05.

^a^ n (%); median (IQR).

Among this subpopulation, there were 2 instances of partial necrosis, 2 partial wound breakdowns, 1 hematoma, 1 wound infection, and 1 fistula. No surgical complication was significantly more common with either PFF technique (Table 4).

**Table 4:** Outcomes in the 2‐Stage Versus 3‐Stage Cartilage Graft Subgroup

Outcome	Overall (N = 29^a^)	2‐stage (N = 16^a^)	3‐stage (N = 13^a^)	*P*
**Surgical complications**				
Hematoma	1 (3.4%)	0 (0%)	1 (7.7%)	.4
Infection	1 (3.4%)	1 (6.2%)	0 (0%)	>.9
Wound breakdown	2 (6.9%)	2 (12%)	0 (0%)	.5
Necrosis	2 (6.9%)	0 (0%)	2 (15%)	.2
Fistula	1 (3.4%)	1 (6.2%)	0 (0%)	>.9
**Cosmetic complications**				
Pincushioning	6 (21%)	2 (12%)	4 (31%)	.4
Hypertrophic scarring	5 (17%)	3 (19%)	2 (15%)	>.9
Excess edema	1 (3.4%)	0 (0%)	1 (7.7%)	.4
Dyspigmentation	4 (14%)	0 (0%)	4 (31%)	**.03**
Trapdoor deformity	0 (0%)	0 (0%)	0 (0%)	‐
Alar notching	5 (17%)	2 (12%)	3 (23%)	.6
**Additional procedures**				
Steroid injection	4 (14%)	1 (6.2%)	3 (23%)	.3
CO_2_ laser	4 (14%)	3 (19%)	1 (7.7%)	.6
Subsequent revision	13 (45%)	6 (38%)	7 (54%)	.4

^a^ n (%).

Pincushioning was the most common cosmetic complication, occurring in 6 patients (21%) in this subanalysis: 2 (12%) in the 2‐stage group and 4 (31%) in the 3‐stage group (*P* = .4). No significant differences were seen between the 2 groups with respect to hypertrophic scaring, excessive edema, trapdoor deformity, or alar notching. There were 4 instances of dyspigmentation noted in the 3‐stage group (31%); this was significantly higher than in the 2‐stage group (0%; *P* = .03). A notable proportion of the overall cartilage grafting subpopulation underwent revision (45%), although this was not significantly more common between the 2 treatment groups. No significant differences were seen between groups with respect to postoperative steroid injections or laser resurfacing.

### Accelerated Takedown

Among the 39 patients who underwent 2‐stage PFF, 7 (18%) were managed with accelerated takedown. These patients were all male. No other significant differences in demographics, comorbidities, or surgical risk factors, including smoking status, radiation exposure, and presence of vascular disease, were seen between these patients and the 32 who underwent standard takedown (Table 5). Eighty‐six percent of the accelerated takedown group had hypertension (vs. 56% in the standard‐takedown group; *P* = .2), 71% were active or former smokers (vs. 88%; *P* = .29), and 14% underwent adjuvant radiation after PFF (vs. 28%; *P* = .7). The groups were not significantly different in defect complexity, size, or location.

**Table 5:** Demographics and Comorbidities in 2‐Stage Group by Takedown Time

Characteristic	Overall (N = 39^a^)	Pedicle takedown ≤21 d (N = 7^a^)	Pedicle takedown >21 d (N = 32^a^)	*P*
**Demographics**				
Age	70 (64, 75)	72 (69, 74)	70 (64, 75)	.6
Gender				.04*
Female	14 (36%)	0 (0%)	14 (44%)	
Male	25 (64%)	7 (100%)	18 (56%)	
**Comorbidities**				
Coronary artery disease	7 (18%)	1 (14%)	6 (19%)	>.9
Hypertension	24 (62%)	6 (86%)	18 (56%)	.2
Diabetes	6 (16%)	0 (0%)	6 (19%)	.6
Chronic kidney disease	6 (15%)	1 (14%)	5 (16%)	>.9
Alcohol use	27 (75%)	5 (71%)	22 (76%)	>.9
Tobacco use				.4
Current	12 (31%)	1 (14%)	11 (34%)	
Former	21 (54%)	4 (57%)	17 (53%)	
Never	6 (15%)	2 (29%)	4 (12%)	
**Treatments/medications**				
Prior head/neck surgery	9 (23%)	0 (0%)	9 (28%)	.2
Prior head/face Mohs	10 (26%)	2 (29%)	8 (25%)	>.9
Prior nasal/facial trauma	3 (8.1%)	0 (0%)	3 (10%)	>.9
Prior head/neck radiation	6 (15%)	0 (0%)	6 (19%)	.6
Adjuvant radiation	10 (26%)	1 (14%)	9 (28%)	.7
**Defect characteristics**				
Defect size, cm^2^	8 (5, 13)	5 (3, 6)	9 (5, 13)	.05
Presence of cartilage defect	17 (44%)	4 (57%)	13 (41%)	.7
Presence of internal nasal defect	20 (51%)	3 (43%)	17 (53%)	.7
Additional reconstruction required	20 (51%)	4 (57%)	16 (50%)	>.9
Defect involvement				
Nasal	39 (100%)	7 (100%)	32 (100%)	‐
Periorbital	11 (28%)	0 (0%)	11 (34%)	.2
Other facial	11 (28%)	1 (14%)	10 (31%)	.6

^a^ n (%); median (IQR).

There were no significant differences in rates of complication between the groups. With accelerated takedown, there were no instances of wound breakdown, partial or full flap necrosis, or fistulization, although 1 patient developed a postoperative donor‐site hematoma. Hypertrophic scarring was the most common cosmetic complication, occurring in 14% of the accelerated takedown group and 19% of the standard timeline group (*P* > .9). There were no significant differences between the 2 groups with respect to subsequent revision, laser resurfacing, or steroid injections. Table 6 summarizes the outcomes of this subanalysis.

**Table 6:** Outcomes in 2‐Stage Group by Takedown Time

Outcome	Overall (N = 39^a^)	Pedicle takedown ≤21 d (N = 7^a^)	Pedicle takedown >21 d (N = 32^a^)	*P*
**Surgical complications**				
Hematoma	1 (2.6%)	1 (14%)	0 (0%)	.2
Infection	1 (2.6%)	0 (0%)	1 (3.1%)	>.9
Wound breakdown	3 (7.7%)	0 (0%)	3 (9.4%)	>.9
Necrosis	0 (0%)	0 (0%)	0 (0%)	‐
Fistula	1 (2.6%)	0 (0%)	1 (3.1%)	>.9
**Cosmetic complications**				
Pincushioning	3 (7.7%)	0 (0%)	3 (9.4%)	>.9
Hypertrophic scarring	7 (18%)	1 (14%)	6 (19%)	>.9
Excess edema	2 (5.1%)	0 (0%)	2 (6.2%)	>.9
Dyspigmentation	1 (2.6%)	0 (0%)	1 (3.1%)	>.9
Trapdoor deformity	1 (2.6%)	1 (14%)	0 (0%)	.2
Alar notching	2 (5.1%)	0 (0%)	2 (6.2%)	>.9
**Additional procedures**				
Steroid injection	6 (15%)	1 (14%)	5 (16%)	>.9
CO_2_ laser	10 (26%)	4 (57%)	6 (19%)	.06
Subsequent revision	12 (31%)	3 (43%)	9 (28%)	.7

^a^ n (%).

Four accelerated takedown patients (57%) also received cartilage grafting. None of these patients developed partial or full flap failure, wound breakdown, or any other surgical complications. One patient in this group developed hypertrophic scarring, but no other measured cosmetic complications, including alar notching, were observed in this subgroup. With respect to revision rates, one of these patients went on to receive both CO_2_ laser resurfacing and subsequent revision debulking, while another one each received either laser or debulking.

### Predicting Complications

Univariate logistic regression identified female gender as a predictor of surgical complication, although this association did not maintain significance in a multivariable model accounting for age, defect complexity, and treatment technique. Patients pursuing revision surgery were younger on average than those who did not; this difference approached but did not reach statistical significance (65.9 vs. 71.6; *P* = .07). No other variable was found to independently predict complication or revision.

## Discussion

In the present cohort, the 2‐stage PFF technique was not associated with greater rates of surgical complications, cosmetic complications, or revision than the 3‐stage technique. In fact, 3‐stage PFFs were associated with significantly higher rates of postoperative dyspigmentation. While there were no differences in comorbidities, surgical risk factors, or defect size between groups, the 3‐stage group did contain a higher proportion of patients with cartilage defects, suggesting that some of these findings may have been due to differential defect complexity. Nonetheless, the efficacy and safety of 2‐stage PFFs is apparent.

The relative overrepresentation of cartilage defects in the 3‐stage group is in accordance with the conventional wisdom that more complex reconstructions warrant the more robust 3‐stage technique.^4^, ^5^, ^9^ The 3‐stage technique is thought to provide maximal blood flow to the recipient site and grant the surgeon an intermediate stage to safely contour cartilage grafts without threatening the reconstruction's vascularity.^9^ Indeed, in their 2015 retrospective study of 2‐stage versus 3‐stage PFFs, Santos Stahl et al found significantly more complex reconstructions, judged by the number of full‐thickness defects, additional procedures required such as cartilage grafting and inner nasal lining reconstruction, and the number of previous operations, in their 3‐stage group.^5^

The present results also challenge this heuristic. Two‐stage PFFs were not associated with poorer cosmetic or surgical outcomes in the subanalysis of patients requiring cartilage grafting. Additionally, there were no significant differences in defect size or presence of concomitant through‐and‐through defects between the 2‐ and 3‐stage groups in this subanalysis. Together, these data suggest that the 2‐stage technique can reliably support complex, cartilaginous reconstructions. This finding supports results from the Santos Stahl et al cohort, where no significant difference in rates of partial flap necrosis was seen between complex defects reconstructed in 2 versus 3 stages.^3^, ^5^

In a separate study, the Stahl group investigated cosmetic outcomes in 2‐ and 3‐stage nasal reconstructions.^6^ Overall, the 3‐stage technique did not produce superior cosmesis, and in the case of thickness at the nasal ala, it actually performed poorer than the 2‐stage technique, even when controlling for defect complexity and the presence of cartilage grafting.^6^ The authors hypothesized that increased fibrosis secondary to additional surgical stages might explain this difference, contrasting conventional wisdom that the 3‐stage approach allows more aggressive thinning.^3^, ^6^ In the present cohort, no significant differences were seen between the 2‐ and 3‐stage approach in the general analysis and in the cartilage grafting subanalysis with respect to postoperative revision or debulking procedures, again challenging the idea of superior thinning with the 3‐stage approach.

The Stahl study also noted that donor‐site complaints, including scar pain, itching, and dysesthesia, were more common in the 3‐stage group than the 2‐stage group.^6^ The present investigation and these antecedent studies must be considered critically in order to optimize the experience of patients undergoing PFF. The 2‐stage technique reduces potentially risky surgical episodes, time spent in cosmetically poor intermediate stages, and possibly also donor‐site morbidity.^3^, ^5^, ^6^ If outcomes are not inferior with this technique, perhaps it should be offered to patients as a first choice, even in cases of complex reconstruction.

However, elsewhere in the literature, the cosmetic superiority of the 3‐stage technique has been upheld. Lo Torto et al, for instance, observed in their population of high‐vascular‐risk patients improved patient‐ and provider‐judged cosmetic outcomes for those treated in 3 stages as opposed to 2.^10^ Vascular risk factors were present in both groups in our cohort, and we did not observe a similar correlation; however, our outcome measures differed from the Lo Torto group.

Regarding the 2‐stage approach, there is a growing body of literature suggesting that early pedicle division is safe and effective. In their 2011 work, Somoano et al compared a group of 26 PFFs with early pedicle takedown (mean 7.2 days) with a group of 9 conventional PFFs.^7^ No patients in the accelerated takedown group had flap failure or other significant surgical complications, including 5 patients who also had cartilage grafting. Telephone interviews showed that patients preferred early takedown. While there was a notable prevalence of tobacco use, hypertension, and other vascular risk factors in their accelerated takedown group, the proportion was significantly higher in the conventional takedown group, leading the authors to conclude that pedicle division at 1 week is safe in younger patients with fewer risk factors.^7^ Other investigators have used imaging techniques, such as indocyanine green angiography and cutaneous ultrasound, to support the safety of accelerated pedicle takedown as early as postoperative day 7 without increased complications.^11^, ^12^ One study utilizing cutaneous ultrasound demonstrated that the average time for the PFF to obtain autonomous blood flow at the recipient site was 6.6 days.^11^

The present study supports this promising role of early pedicle division. No significant differences in surgical complications, cosmetic complications, or revision rates were seen between the accelerated and standard takedown groups. There were no flap failures with accelerated takedown, including among those patients who also received cartilage grafting. Additionally, among patients who received both cartilage grafting and accelerated takedown, no alar notching was seen, suggesting this technique adequately supported the cartilaginous reconstruction, at least at the ala. Notably, while gender was distributed asymmetrically between groups, those receiving accelerated takedown in this study otherwise were not significantly healthier or less risky than those managed with standard timelines, and neither group contained significantly more complex defects. As with the noninferiority of 2‐stage PFFs compared to 3‐stage, accelerated pedicle takedown, if surgically, cosmetically, and functionally non‐inferior to standard takedown, promises to improve patient well‐being by further reducing the time spent in intermediate stages.

The inverse relationship noted between age and revision, while not statistically significant in this study, agrees with existing literature. Younger patients have demonstrated poorer aesthetic satisfaction leading to increased revision rates after facial skin cancer resection, Mohs surgery, and free flap reconstruction.^13^, ^14^, ^15^ With this in mind, younger patients may benefit from focused preoperative counseling.

### Limitations

Given the retrospective design of this study, documentation was not uniform across the cohort in factors such as defect size. Additionally, there were no consistent criteria for assigning patients to treatment groups; this was instead guided by surgeon preference (which may have been influenced in part by defect complexity), patient preference, and other individualized clinical factors. For instance, 2 patients in our cohort were scheduled for second‐stage pedicle takedown but had complications noted at that time and ultimately were managed in 3 stages. Together, this introduces some selection bias that could be avoided with a prospective design.

A prospective design could also ensure equal application of the 2 PFF techniques to the spectrum of defect complexity. While the 2‐ and 3‐stage cohorts were balanced in terms of major surgical risk factors, the 3‐stage group in this study did contain a higher proportion of cartilage defects. Our results may have been different if the 2‐stage technique had been applied to an equal proportion of these defects. However, we remain encouraged by the present results as the 2‐stage technique was not inferior in the cartilage grafting subanalysis, where defect complexity was statistically balanced.

We did not record the specific subunits involved in patients' defects or the exact site of complication. Given prior literature demonstrating different donor site morbidity for 2‐ and 3‐stage PFFs, outcomes in our cohort included both the donor and recipient sites. Capturing data on a more granular level will allow future studies to assess the performance of PFF by location and avoid potential confounding in cases where additional reconstructions, such as cheek advancements, are used. Furthermore, revision procedures in our analysis did not include isolated functional surgery, such as dacryocystorhinostomy for iatrogenic epiphora. Including functional measures and expanding complications captured in future studies will further elucidate the role of each technique.

Finally, the sample size limits the ability to definitively show no difference between techniques. Assuming a complication rate of approximately 20% and an intergroup difference of 10% clinically negligible, a future study would need to include 398 patients to achieve 80% power at a significance level of 5%. This number grows as the expected difference shrinks. Expanded sample sizes will be important to establish if outcomes are truly equivalent.

## Conclusion

In conclusion, interrogating our institution's cohort of PFFs revealed that both 2‐ and 3‐stage PFF techniques are safe and effective in midface reconstruction. Patients with complex defects requiring cartilaginous reconstruction can be successfully managed in 2 stages without compromising aesthetics or risking increased complication rates. In the 2‐stage group, accelerated pedicle takedown was not associated with increased complications. The cohorts were notably statistically balanced in terms of major risk factors such as smoking status and radiation exposure. In the context of clinical practice, this study supports accelerated takedown in well‐selected patients.

## Author Contributions

**W. Jack Palmer**, design, conduct, analysis, and presentation of research; **Dev Amin**, design, conduct, analysis, and presentation; **Praneet Kaki**, conduct, analysis, and presentation; **Matt Davis**, conduct and presentation; **Eric Fei**, conduct and presentation; **Neha Garg**, conduct and presentation; **Daniel J. Campbell**, analysis and presentation; **Dana Michlin**, presentation; **Khashayar Arianpour**, analysis and presentation; **Howard Krein**, design, conduct, analysis, and presentation; **Ryan Heffelfinger**, design, conduct, analysis, and presentation.

## Disclosures

### Competing interests

None.

### Funding

None.
